# A FRAMEWORK FOR IDENTIFYING MAJOR RESEARCH PRIORITIES: THE CITRA RESEARCH-TO-PRACTICE CONSENSUS WORKSHOP MODEL

**DOI:** 10.1093/geroni/igac059.284

**Published:** 2022-12-20

**Authors:** Karl Pillemer

**Affiliations:** Cornell University, Ithaca, New York, United States

## Abstract

The CITRA Research-to-Practice Consensus Workshop Model is an evidence-based method for identifying research priorities on aging-related topics. Its major goals are to promote knowledge translation and equal-status dialogue among stakeholders from multidisciplinary backgrounds. In this presentation, Dr. Pillemer, co-creator of the CITRA model, will provide an overview of the 5-step method: (1) Identifying a topic that is both an important challenge in aging-related policy or practice and one on which there is scientific evidence; (2) Selecting a panel of multidisciplinary experts from relevant stakeholder groups; (3) Preparing a non-technical background document that summarizes available research findings; (4) Convening multidisciplinary experts in a consensus conference involving panel presentations, discussion of the background document, and consensus activities; and (5) Preparing a final consensus report and disseminating the findings to relevant policy organizations, practice groups, and funding agencies. Common challenges and practical tips for executing each of the 5 steps will be addressed.

